# Taxonomically Restricted Genes Are Associated With Responses to Biotic and Abiotic Stresses in Sugarcane (*Saccharum* spp.)

**DOI:** 10.3389/fpls.2022.923069

**Published:** 2022-06-30

**Authors:** Cláudio Benício Cardoso-Silva, Alexandre Hild Aono, Melina Cristina Mancini, Danilo Augusto Sforça, Carla Cristina da Silva, Luciana Rossini Pinto, Keith L. Adams, Anete Pereira de Souza

**Affiliations:** ^1^Center of Molecular Biology and Genetic Engineering (CBMEG), University of Campinas (UNICAMP), Campinas, Brazil; ^2^Department of Botany, University of British Columbia, Vancouver, BC, Canada; ^3^Agronomy Department, Federal University of Viçosa (UFV), Viçosa, Brazil; ^4^Sugarcane Research Advanced Centre, Agronomic Institute of Campinas (IAC/APTA), Ribeirão Preto, Brazil; ^5^Institute of Biology, University of Campinas (UNICAMP), Campinas, Brazil

**Keywords:** orphan genes, sugarcane hybrid, stress condition, RNA-Seq, gene expression

## Abstract

Orphan genes (OGs) are protein-coding genes that are restricted to particular clades or species and lack homology with genes from other organisms, making their biological functions difficult to predict. OGs can rapidly originate and become functional; consequently, they may support rapid adaptation to environmental changes. Extensive spread of mobile elements and whole-genome duplication occurred in the *Saccharum* group, which may have contributed to the origin and diversification of OGs in the sugarcane genome. Here, we identified and characterized OGs in sugarcane, examined their expression profiles across tissues and genotypes, and investigated their regulation under varying conditions. We identified 319 OGs in the *Saccharum spontaneum* genome without detected homology to protein-coding genes in green plants, except those belonging to Saccharinae. Transcriptomic analysis revealed 288 sugarcane OGs with detectable expression levels in at least one tissue or genotype. We observed similar expression patterns of OGs in sugarcane genotypes originating from the closest geographical locations. We also observed tissue-specific expression of some OGs, possibly indicating a complex regulatory process for maintaining diverse functional activity of these genes across sugarcane tissues and genotypes. Sixty-six OGs were differentially expressed under stress conditions, especially cold and osmotic stresses. Gene co-expression network and functional enrichment analyses suggested that sugarcane OGs are involved in several biological mechanisms, including stimulus response and defence mechanisms. These findings provide a valuable genomic resource for sugarcane researchers, especially those interested in selecting stress-responsive genes.

## Introduction

Recent advances in sugarcane genomics have created opportunities to systematically reveal the evolutionary history and diversification of the *Saccharum* group. However, the complexity of the sugarcane genome, mainly due to its size, ploidy level, and large number of mobile elements (Thirugnanasambandam et al., 2018), has hindered advances in the genomics of this important crop species. Despite the economic importance of sugarcane due to its use as a source of sugar, biofuel, and fibre, its reference genomes, including a chromosome-level *Saccharum spontaneum* genome (Zhang et al., 2018), a monoploid genome from the R570 variety (Garsmeur et al., 2018), and an SP80-3280 hybrid genome (Souza et al., 2019), have been only recently reported.

Two events of whole-genome duplication (WGD) are thought to have occurred during the evolution of the *Saccharum* group (Ming et al., 1998; Paterson et al., 2012). WGD is a major mechanism responsible for species diversification and adaptation (Soltis et al., 2009; Renny-Byfield and Wendel, 2014). These recent events of polyploidization occurring within the Saccharinae group provide an opportunity to investigate the fate of duplicated genes. Genome duplication initially results in gene duplication and gene redundancy. After duplication, some gene copies preserve their original function, while most of them are eliminated through negative selection (Tautz and Domazet-Lošo, 2011). However, some copies under positive selection, after sequence diversification, may acquire a new biological function (Van de Peer et al., 2009). Divergence of pre-existing genes is one of the mechanisms underlying the emergence of new genes (Tautz and Domazet-Lošo, 2011). An alternative origin has been proposed: new genes originate from a non-coding sequence (Singh and Syrkin Wurtele, 2020; Vakirlis et al., 2020).

Taxonomically restricted, lineage-specific or orphan genes (OGs), which have no homology to genes in other taxa, may contribute to evolutionary novelties and might be responsible for some lineage-specific trait origins (Wilson et al., 2005; Khalturin et al., 2009; Tautz and Domazet-Lošo, 2011). Even though we have not given sufficient attention to these genes, comparative genomic studies have estimated that OGs constitute at least 1% of the total genes in a genome, depending on the alignment rate and taxonomic level considered (Khalturin et al., 2009; Arendsee et al., 2014; Prabh and Rödelsperger, 2016). Several studies have been carried out to characterize OGs in plants at the species level: Arabidopsis (Li and Wurtele, 2014), sweet orange (Xu et al., 2015), rice (Guo et al., 2007), moso bamboo (Zhang et al., 2022), and at the family level: Brassicaceae (Donoghue et al., 2011) and Poaceae (Campbell et al., 2007). However, there is limited information about the function of most of these OGs, as they lack recognizable domains and functional motifs.

OGs are known to play a role in primary metabolism and response to environmental changes in plants. By establishing a gene-editing system, Jiang et al. (2020) revealed that an orphan gene (*BrOGs*) in *Brassica napus* plays a vital role in soluble sugar metabolism. In *Arabidopsis*, a functional analysis of the well-studied orphan gene *QQS* (*qua quine starch*) indicated that it could act in regulation of nitrogen allocation, affecting the protein content (O’Conner et al., 2018). Additionally, there are several works showing that OGs are regulated in response to biotic and abiotic stresses (Beike et al., 2014; Giarola et al., 2014; Khraiwesh et al., 2015; Schlötterer, 2015; Kaur et al., 2017). For example, an OG named *TaFROG* enhanced wheat resistance to Fusarium head blight (Perochon et al., 2015) and an OG in *Vigna unguiculata* (*UP12_8740*) increased plant tolerance to osmotic stresses and soil drought (Li et al., 2019). A *Physcomitrium patens* OG (*PpARDT*) was functionally characterized, and knockout mutant displayed reduced drought tolerance (Dong et al., 2022).

Despite the biological relevance of these taxonomically restricted genes, no previous reports described their occurrence and expression profile in the *Saccharum* complex. To advance our knowledge about OGs in sugarcane, a comparative genomic approach is needed for their identification, followed by regulatory inference based on gene expression analysis. In this study, we identified and characterized sugarcane OGs and their expression patterns across tissues and genotypes. Additionally, we analysed expression data from different conditions to identify those under which these genes are positively or negatively regulated in sugarcane.

## Materials and Methods

### Orphan Gene Identification

A phylostratigraphic approach based on a sequence homology search was used to identify OGs in the sugarcane genome (Domazet-Lošo et al., 2007; McLysaght and Hurst, 2016). These analyses rely on sequence alignments to detect genes that lack homology in a focal species in comparison with a target clade. For this analysis, we used the gene model from *S. spontaneum* (Zhang et al., 2018) as a reference. First, the protein and coding DNA sequence (CDS) files containing the set of sugarcane genes were filtered using the CD-HIT package v.4.8.1 (Fu et al., 2012); a similarity threshold of 90% was applied for both the CD-HIT and CD-HIT-EST algorithms, which were employed for the protein and CDS files, respectively. This step was performed to remove redundancies in the dataset once all the homologous genes and duplications were included in the annotated sugarcane genome. Subsequently, a series of local alignments using both the sugarcane proteome and CDSs were performed to remove genes with homology in other species. First, to reduce the subset of candidate genes, we filtered out all sugarcane genes with detected homology to genes annotated in 13 angiosperm species including seven Poaceae species (*Arabidopsis thaliana* TAIR10, *Brachypodium distachyon* v3.1, *Citrus sinensis* v3.1, *Eucalyptus grandis* v2.0, *Miscanthus sinensis* 7.1, *Oryza sativa* 7.0*, Phaseolus vulgaris* v2.1*, Panicum virgatum* v4.1, *Setaria italica* v2.2*, Solanum lycopersicum* ITAG3.2*, Sorghum bicolor* v3.1.1*, Oropetium thomaeum* v1.0, and *Zea mays* 284 v6). The annotated sequences were downloaded from Phytozome v.13 (Goodstein et al., 2011) and converted into a database. The remaining subset of sugarcane genes was aligned to non-redundant proteins (NR) and nucleotides (NT) from the National Center for Biotechnology Information (NCBI) database, and genes without homology in previous filtering steps were discarded. In all filtering steps, we used BLASTp and BLASTn to detect homology at the protein and nucleotide levels, respectively. All results were generated using a permissive E-value cut-off ≤10–6, allowing more relationships to be detected and increasing the chance of selecting a real OG. To discard the hypothesis that predicted OGs were missing from the genome annotation, we mapped each OG back on the chromosomes of seven species downloaded from Phytozome v.13 (*A. thaliana*, *Panicum hallii*, *S. italica*, *Z. mays*, *S. bicolor*, *S. spontaneum*, and *M. sinensis*) using sim4 software, which employs a splice-aware alignment method (Florea et al., 1998).

### Orphan Gene Characteristic Features and Sequence Homology

The FASTA files containing sugarcane chromosome information and gff3 files were used for manual curation of the OGs. The position of each OG exon was used as a starting point to check intron/exon boundaries as well as the presence of start and stop codons using Artemis software v.18.0 (Carver et al., 2011). To characterize the physical and chemical properties of sugarcane genes (OGs and non-OGs), we calculated protein parameters [protein length, molecular weight, the instability index, hydrophobicity, the isoelectric point, and the grand average of hydropathicity (GRAVY)] using ProtParam tools implemented in the Bio.SeqUtils package, a Biopython module (Cock et al., 2009). To assess the protein-coding potential of these genes, we estimated the probability of each OG being a coding RNA using Coding Potential Calculator (CPC2; Kang et al., 2017). Additionally, all predicted OGs were aligned to the non-coding RNA databases derived from Rfam, Tair, Ensemble long non-coding RNAs (lncRNAs), CANTATA (Szcześniak et al., 2015), and GreeNC (Paytuví Gallart et al., 2015) using nhmmer with an E-value parameter ≤0.001 (Wheeler and Eddy, 2013). We checked for transposable element (TE) insertion in the OG sequences by using a reference collection of transposons from the Repbase database (Bao et al., 2015) using CENSOR (Jurka et al., 1996). To search for homologues within the Saccharinae subtribe, we aligned predicted proteins of OGs to annotated proteins from the draft genomes of *Saccharum* hybrids SP803280 (Souza et al., 2019) and R570 (Garsmeur et al., 2018) and complete genomes from *M. sinensis* (Mitros et al., 2020) and *S. spontaneum* (Zhang et al., 2018) using BLASTp with an E-value ≤10^−6^.

### RNA-Seq Experimental Data: Retrieval and Pre-processing

An extensive search for papers reporting RNA-Seq data in sugarcane was performed, followed by a search of the NCBI Sequence Read Archive (SRA) repository (Supplementary Table 1). The selected RNA-Seq samples were retrieved using the ‘fastq-dump’ program from the SRA toolkit (version 8.22), and SRA files were converted to fastq-format files. The raw reads were subjected to quality control using Trimmomatic v0.36 (Bolger et al., 2014) to remove adapter and low-quality sequences. Three reference transcriptomes, including two full-length transcriptomes, were also selected to confirm the selected OGs being transcribed. In the first set of IsoSeq data, RNA samples were extracted from the top and bottom internodes of 22 genotypes (Hoang et al., 2017), and in the second set, RNA samples were obtained from leaves of a commercial sugarcane variety from Thailand (Piriyapongsa et al., 2018). The third transcriptome, which was *de novo* assembled from short reads, was extracted from the leaves of six sugarcane hybrids (Cardoso-Silva et al., 2014). A local alignment using the BLASTn program was performed with an e-value cut-off ≤1 × 10^−6^ to infer the homology of putative OGs to sugarcane transcripts.

### Orphan Gene Expression Profile and Differential Expression

RNA-Seq libraries were constructed for two purposes: (i) to unveil the expression patterns of OGs across sugarcane tissues and genotypes and (ii) to identify DE OGs, especially under stress conditions. The expression level of each gene was estimated by mapping the transcriptomes against the whole gene set of sugarcane using Salmon (Patro et al., 2017). The expression level of a given gene was calculated by the log transformation method implemented in Salmon [transcripts per million (TPM)], which represents the relative abundance of a transcript among a population of transcripts. Heatmaps representing the expression levels of OGs in experiments testing for differential gene expression and evaluating expression across tissues/genotypes were created using the ‘superheat’ R package (Barter and Yu, 2018).

To investigate whether OGs were DE, we designed RNA-Seq experiments including biotic and abiotic stresses, developmental stages, and sucrose accumulation. Cleaned reads from each library originating from experiments with biological replicates were mapped to the complete set of sugarcane genes (CDS FASTA format) using Salmon v.0.12.0 to quantify transcript abundance (Patro et al., 2017). The DESeq2 package v.3.9 (Love et al., 2014) was used to predict DEGs in each experiment using the raw read counts as input data. In cases where the same sample was sequenced in multiple runs, the technical replicates were collapsed before starting the DEG analysis. To minimize quantification biases, genes with fewer than 10 reads mapped per sample were filtered out before the gene expression analysis. The DEGs were estimated assuming a negative binomial distribution for each gene, applying a function that estimates the size factor and reducing bias caused by library size (normalization by the median ratio; gene count divided by the sample size). A value of *p* < 0.05 and an absolute log2 fold change ≥2 were used as thresholds for determining whether genes were apparently DE. For each predicted OG, hypothesis testing was performed, in which the null hypothesis was no differences between the control and treatment groups, thus supporting the assumption that any difference in gene expression occurred merely by chance.

### Orphan Gene Co-expression Network and Functional Annotation

The expression matrix of the 218 samples across sugarcane tissues and genotypes was used to build a co-expression network with the ‘WGCNA’ R package (Langfelder and Horvath, 2008). A weighted adjacency matrix was constructed using pairwise Pearson’s correlation coefficient measures and an estimated power threshold for scale-free independence (*R*^2^ > 0.8 and largest mean connectivity). Subsequently, the calculated matrix was converted to a topological overlap matrix (TOM), which evaluates gene pair correlations and the degree of agreement with other genes in the matrix (Yip and Horvath, 2007). After that, we inferred network functional modules by employing average linkage hierarchical clustering in accordance with the TOM-based dissimilarity measure. We used a soft threshold power of 7 (*R*^2^ of 0.81 and mean connectivity of 164) to calculate the TOM. Clusters were defined according to a hierarchical dendrogram using adaptive branch pruning as implemented in the ‘dynamicTreeCut’ R package (Langfelder et al., 2007). We conducted functional enrichment analysis of the modules containing OGs based on Gene Ontology terms. Then, we assumed a guilty-by-association approach to obtain some insight into OG functionality. Additionally, we checked for OG similarity with known protein domains using hmmscan from the HMMER3 suite (Finn et al., 2011), aligning the domains to the Pfam v35 database (Mistry et al., 2020).

## Results

### Identification and Characterization of Sugarcane Orphan Genes

After removing redundancies in the sugarcane gene model, which contained 83,826 genes from *S. spontaneum* (Zhang et al., 2018), we obtained a total of 51,675 NR protein-coding genes. These sugarcane genes were aligned to the proteomes of 13 angiosperm species, including seven Poaceae species, represented by 435,957 proteins. This alignment returned 1,536 sugarcane genes with no homology with any protein represented. Next, these subsets of ‘no-hit’ genes were aligned to the NR protein database, and 442 genes with no homology were found. Finally, these remaining sets were aligned to the NT database. As a result, a total of 335 genes were identified as sugarcane OGs due to their lack of homology to other genes.

Homology searches may fail to detect homologues in other species, resulting in spurious OG prediction. To minimize this effect, we did not rely only on homology searches but also mapped OG CDSs onto each chromosome of seven grass genomes (*S. spontaneum*, *M. sinensis*, *S. bicolor*, *P. hallii*, *Z. mays*, *S. italica*, and *O. sativa*) and the genome of *A. thaliana*. Although OGs were not annotated as complete genes, except in the *Saccharum* group, we found vestiges of exons of these genes in all grass chromosomes. However, we did not detect OG vestiges in *A. thaliana* chromosomes (Supplementary Table 2).

**Figure 1:**
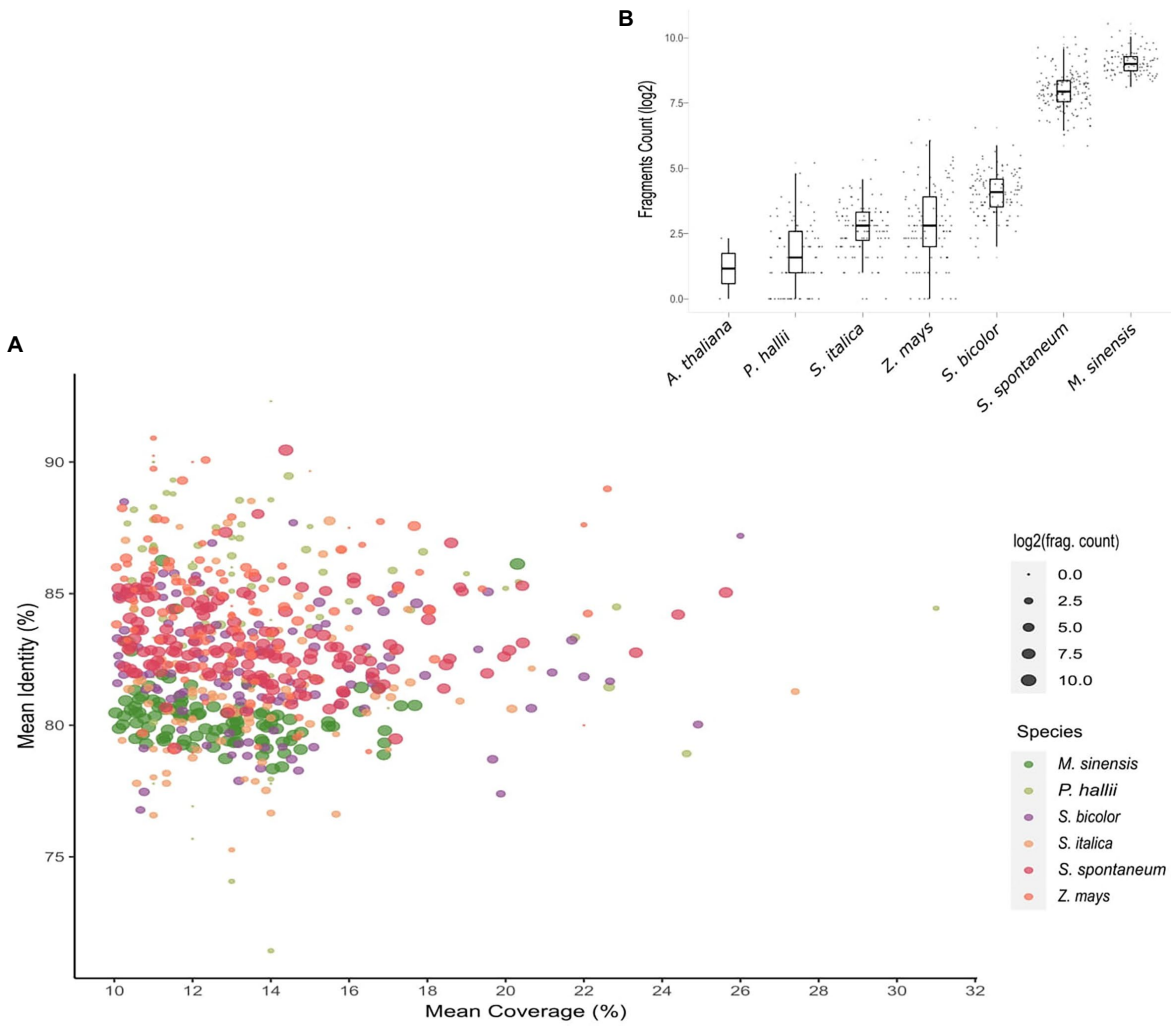
Evidence of orphan gene (OG) vestiges within grass genomes. **(A)** Scatterplot showing the average percentage identity and coverage of each OG in six grass species. Each dot represents an individual OG. The dot sizes represent the number of fragments of each sugarcane OG in other grass species. The number of fragments of each OG in the focal species is also shown in the bar plot **(B)**.

To better understand the distribution of these putative OGs across grass genomes, we assessed chromosome regions in which dispersed fragments of the OGs aligned with at least 10% of their length. Intriguingly, the closer the phylogenetic relationship with *Saccharum* was, the higher the number of OG fragments observed in grass genomes (Figure 1; Supplementary Table 2). The number of OG fragments ranged from 114 in the *O. sativa* genome to 91,379 in the *M. sinensis* genome. If we consider the *S. spontaneum* genome, the number of OG fragments is even larger. Curiously, there are 25 times more OG fragments in *S. spontaneum* and *M. sinensis* chromosomes than in the sorghum genome, which is the closest relative of these Saccharinae species.

To verify whether OGs are evolutionarily conserved within the Saccharinae subtribe, we searched for OGs homologs in the *Saccharum* spp., including the annotated genome of *M. sinensis*. We found 201 OGs with similarity to other sequences predicted to be protein-coding genes in Saccharinae genomes. Most homologs were present in the *Saccharum* hybrid genome; however, we also found 39 homologs genes in the *M. sinensis* genome. Furthermore, 127 OGs have duplicate copies in *S. spontaneum*, annotated as alleles or paralogues (Supplementary Figure 1; Supplementary Table 3). Additionally, because many OGs were not predicted as coding genes in other *Saccharum* genomes, we aligned them to non-coding RNA sequences. However, we did not detect similarity of any OGs with ncRNAs deposited in public databases, including lncRNAs.

Using a vector machine-based classifier named CPC2, 152 OGs were classified as ncRNAs, and 167 OGs were classified as coding RNAs (Supplementary Figure 2; Supplementary Table 4). A total of 89 OGs were predicted as protein-coding genes with a high probability (> = 0.9), while 47 others were classified as lncRNAs. However, short OGs were more likely to be classified as ncRNAs (*R*^2^ = 0.73, value of *p* < 2.2e^−16^; Supplementary Figure 3). A search for protein domains within OG sequences revealed that most OGs do not show similarity with any functional domains deposited in the Pfam database. Based on these searches, we detected only partial local alignment for 30 OGs (BLAST searches produced no significant alignments to domains; E-value ≤1E-3), of which nine were predicted to be domains of unknown function (Supplementary Table 4).

Physical and chemical analyses revealed that OGs and non-OGs were significantly different in all parameters except GC content (Supplementary Figure 4; Supplementary Table 4). OGs are shorter than non-OGs, with average protein lengths of 136 and 450, respectively. The number of exons in OGs varied from 1 to 21 (*x-* = 3.54; *σ* = 2.37), and 57.9% had three exons or fewer (Supplementary Table 4). The average GC contents of orphan and non-OGs were almost identical, at 56.7 and 56.5%, respectively. Most sugarcane genes had a negative GRAVY (grand average of hydropathy) value, i.e., ~81% of the OGs and ~80% of the non-OGs, which supports the protein being hydrophilic.

### TE Fragments in the OGs

The large number of gene fragments found in the Saccharinae genomes may indicate that some of these genes originated from TE duplication. We aligned all the predicted OGs to TE sequences to test the hypothesis that some of the OGs are TEs and to verify whether some of the OGs were derived from TE insertion (Supplementary Table 5). This search was motivated by previous observations suggesting that 51% of the OGs in rice are derived from TEs (Jin et al., 2019). To investigate this hypothesis more deeply and shed light on the origin of these putative lineage-specific genes, we performed an alignment of the initially selected set of genes (335 OGs) against the Repbase TE library.^1^ A total of 153 putative OGs aligned to TEs with significant hits (E-value ≤1E-10). For a subset of these genes (16 OGs), at least 70% of the sequence aligned to TEs. We assumed that these genes were putative TEs, and we did not consider them to be sugarcane OGs. In the remaining subset (319 OGs), some genes had traces of TE insertion into the coding region, albeit with poor alignment. To better understand this result, we estimated the fractions of both OGs and non-OGs in sugarcane with similarity to TEs. Most sugarcane genes had traces of TEs in their coding region, and ~55% of the OGs and ~75% of the non-OGs had at least 10% of their sequence aligning to TEs (Supplementary Figure 5).

### Evidence of Orphan Gene Expression Across Sugarcane Tissues and Genotypes

We searched for evidence that the 319 OGs were being transcribed across several sugarcane tissues and genotypes by aligning them against reference transcriptomes and RNA-Seq libraries. We selected two representative sugarcane IsoSeq datasets (Hoang et al., 2017; Piriyapongsa et al., 2018), a collection of transcripts from six sugarcane varieties (Cardoso-Silva et al., 2014), and 218 RNA-Seq samples from sugarcane hybrids, *Saccharum officinarum,* and *S. spontaneum* (Supplementary Table 1). Evidence of transcription was detected in 89.34% of the OGs (TPM value ≥1), which were expressed in at least one transcriptome experiment or tissue (Supplementary Table 6). Almost one-third of the OGs had an expression level considered low (1 ≤ TPM ≤ 10), while only 6% of them had a value greater than 100 TPM. In fact, when we compared the expression levels of OGs and non-OGs in four sugarcane tissues (Supplementary Figure 6), we found that OGs had proportionally lower expression levels. However, these differences were almost imperceptible in the meristem tissue (bud).

**Figure 2:**
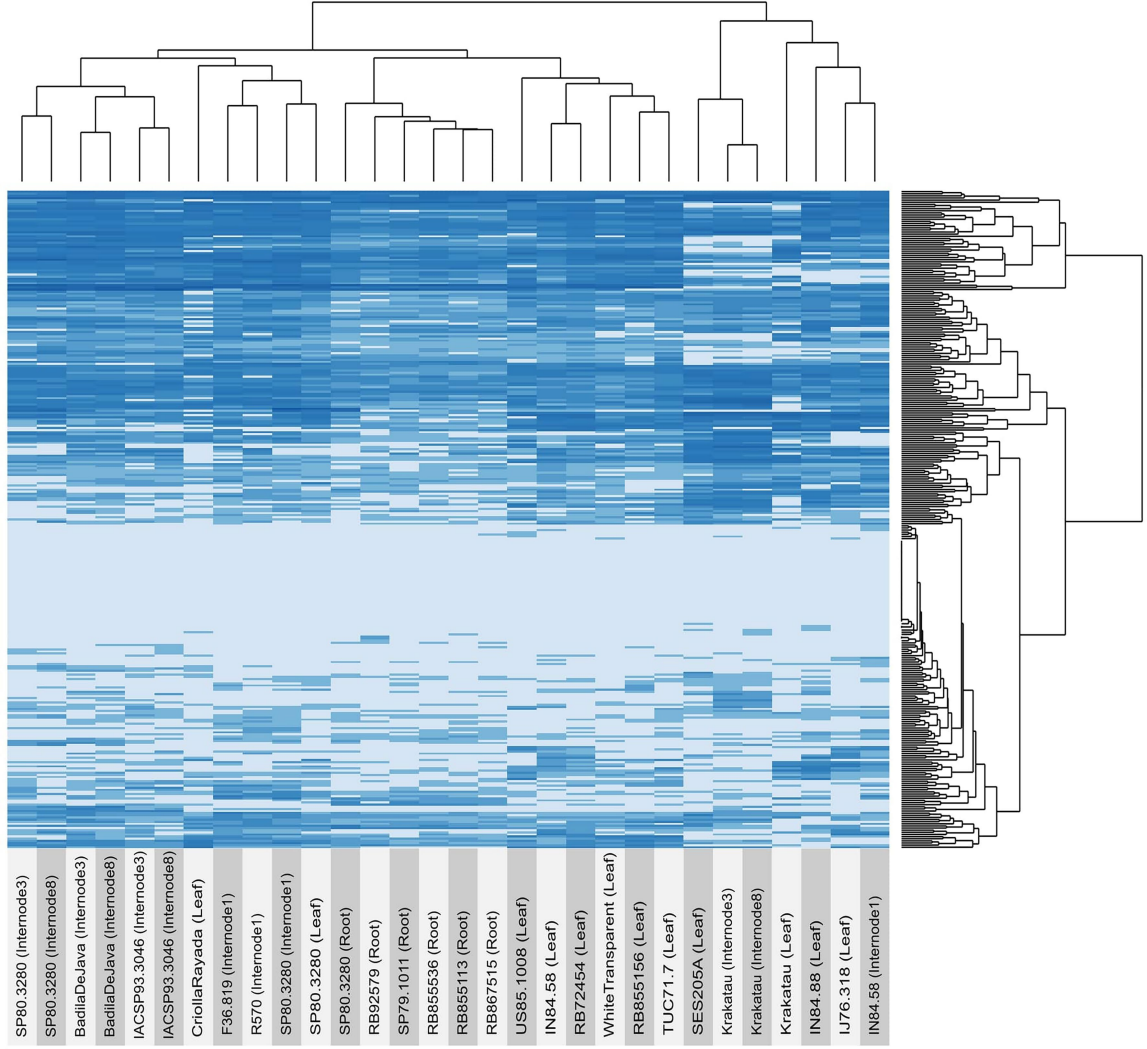
Expression profiles of orphan genes (OGs) in several sugarcane tissues and genotypes. The expression of each gene (TPM) was estimated using a pseudoalignment method implemented in Salmon software.

The expression profile of OGs across tissues and genotypes revealed a clear pattern of sample clustering. Overall, OGs showed similar expression patterns among genotypes when we compared the same tissue (Figure 2 and Supplementary Table 6). Although the expression matrix combined tissues and genotypes, we also observed clustering by genotype to the same degree. For example, samples originating from *S. spontaneum* (Krakatau, IN84_58, and SES205A) were clustered together and separated from those originating from *S. officinarum* (BadilaDeJava, CriollaRayada, and WhiteTransparent) and *Saccharum* hybrids. Notably, samples originating from *Saccharum* hybrids and *S. officinarum* had more similar expression patterns than those originating from *S. spontaneum*. In particular, the expression pattern of OGs in the internodes of *S. spontaneum* was noticeably different from that in *S. officinarum* and hybrids. Furthermore, some OGs presented contrasting expression patterns between samples from *S. officinarum* and *S. spontaneum*. Specifically, a subset of 42 OGs had an average expression level that was three times higher in *S. officinarum* than in *S. spontaneum*. In contrast, the expression level of 35 OGs was higher in *S. spontaneum*. Interestingly, genotypes originating from the same geographical location tended to be clustered together because they had more similar expression profiles. In particular, this pattern was observed in the expression levels of OGs in internode 1 of French hybrids (F36.819 and R570) and the roots of Brazilian hybrids (RB855536, RB855113, and RB867515).

Interestingly, a few OGs seemed to have tissue-specific regulation, as observed for some genes only expressed in roots (Sspon.06G0001310 and Sspon.06G0024430), internode 1 (Sspon.08G0030340), internodes 3 and 8 (Sspon.05G0032460 and Sspon.08G0007980), and all internodes (Sspon.06G.0027080).

### Orphan Genes Are Differentially Expressed Under Stress Conditions

We performed eight RNA-Seq experiments representing a variety of conditions to test whether OGs change their expression levels (Table 1). After filtering the raw data, we selected more than 13 billion high-quality reads to perform gene expression analysis.

**Table 1:** RNA-Seq experiments performed for sugarcane gene expression analysis.

**Accession**	**Condition**	**Cultivar/Species**	**Tissue**	**Rep**^**1**^	**References**
PRJNA474042	yellow canopy syndrome	Hybrid	leaf	5	Marquardt et al., 2019
PRJNA479814	developmental stages	Q208 and KQ228	root/leaf/ internode	3	Thirugnanasambandam et al., 2019
PRJNA483518^*^	cold stress	Guitang08-1,180 and ROC22	leaf	4	Tang et al., 2018
PRJNA533093	low nitrogen	Badila	leaf/root	3	Yang et al., 2019
PRJNA291816	smut disease	RB925345	bud	3	Bedre et al., 2019
PRJNA415122	*S. scitamineum* infection	CP74_2005	bud	3	McNeil et al., 2018
PRJNA371469	osmotic stress	*S. officinarum*	root/leaf	2	Pereira-Santana et al., 2017
PRJNA681593	sucrose accumulation	Hybrids	top and bottom internodes	3	Aono et al., 2021

* Represented by multiple accessions (see more details in Supplementary Table 1).

1 Number of biological replicates.

We observed at least one DE OG in five of the eight RNA-Seq experiments (Supplementary Table 7). We did not detect DE OGs in the RNA-Seq experiments related to developmental stage, sucrose accumulation, and plant infection with *Sporisorium scitamineum*. Generally, more genes were DE in the experiments related to abiotic stress than in those related to biotic stress. For example, we estimated that 6,440 genes were DE under cold stress (in genotypes Guitang08-1,180 and ROC22), while only 1,548 genes were DE after infection with yellow canopy syndrome, and 2,612 genes were DE in plants infected with smut disease. Overall, we identified 66 OGs that were DE under at least one type of stress (log_2_FoldChange ≥ 2; padj < 0.05). Most of the genes were regulated by abiotic stresses, while only 4 OGs were regulated by biotic stresses. We detected only two OGs (Sspon.02G0052680-1C and Sspon.06G0009900-2C) that were DE in both the osmotic stress and cold stress experiments, and one gene (Sspon.04G0024550-1B) showed DE in both the cold stress and low-nitrogen experiments.

**Figure 3:**
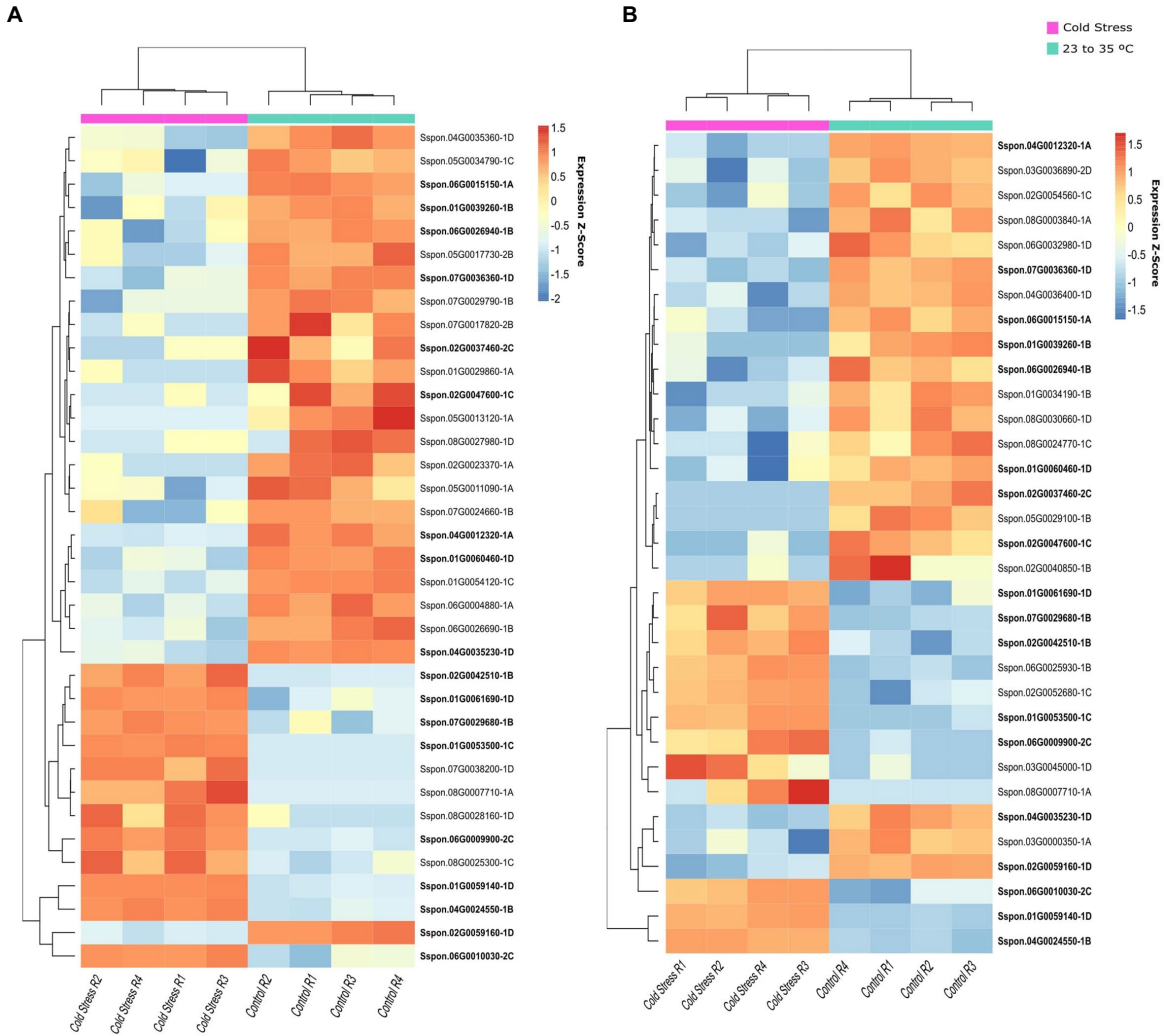
Orphan genes (OGs) differentially expressed (DE) under cold stress. Hierarchical clustering of the genes expressed at normal and cold temperatures with four replicates for each treatment (row). DE analysis was carried out in leaf tissues at an ambient temperature (ranging from 23°C to 35°C) and a cold temperature (4°C in a well-controlled climate chamber) in two sugarcane genotypes: ROC22 **(A)** and Guitang08-1180 **(B)**.

In the cold stress experiment, we identified 8,921 DE genes in the hybrid Guitang08-1,180 (5,644 upregulated and 3,277 downregulated) and 8,913 DE genes in the hybrid ROC22 (5,596 upregulated and 3,317 downregulated), and 72% of these genes were DE in both genotypes. A total of 50 OGs were determined to be DE in this experiment, with 33 OGs in Guitang08-1,180 (12 upregulated and 21 downregulated) and 36 OGs in the ROC22 hybrid (12 upregulated and 24 downregulated), while 18 OGs were DE in both genotypes (Figure 3 and Supplementary Table 7). An OG that was upregulated in both genotypes (Sspon.06G00100300) has three conserved copies annotated in the *S. spontaneum* genome (Supplementary Figure 7).

**Figure 4:**
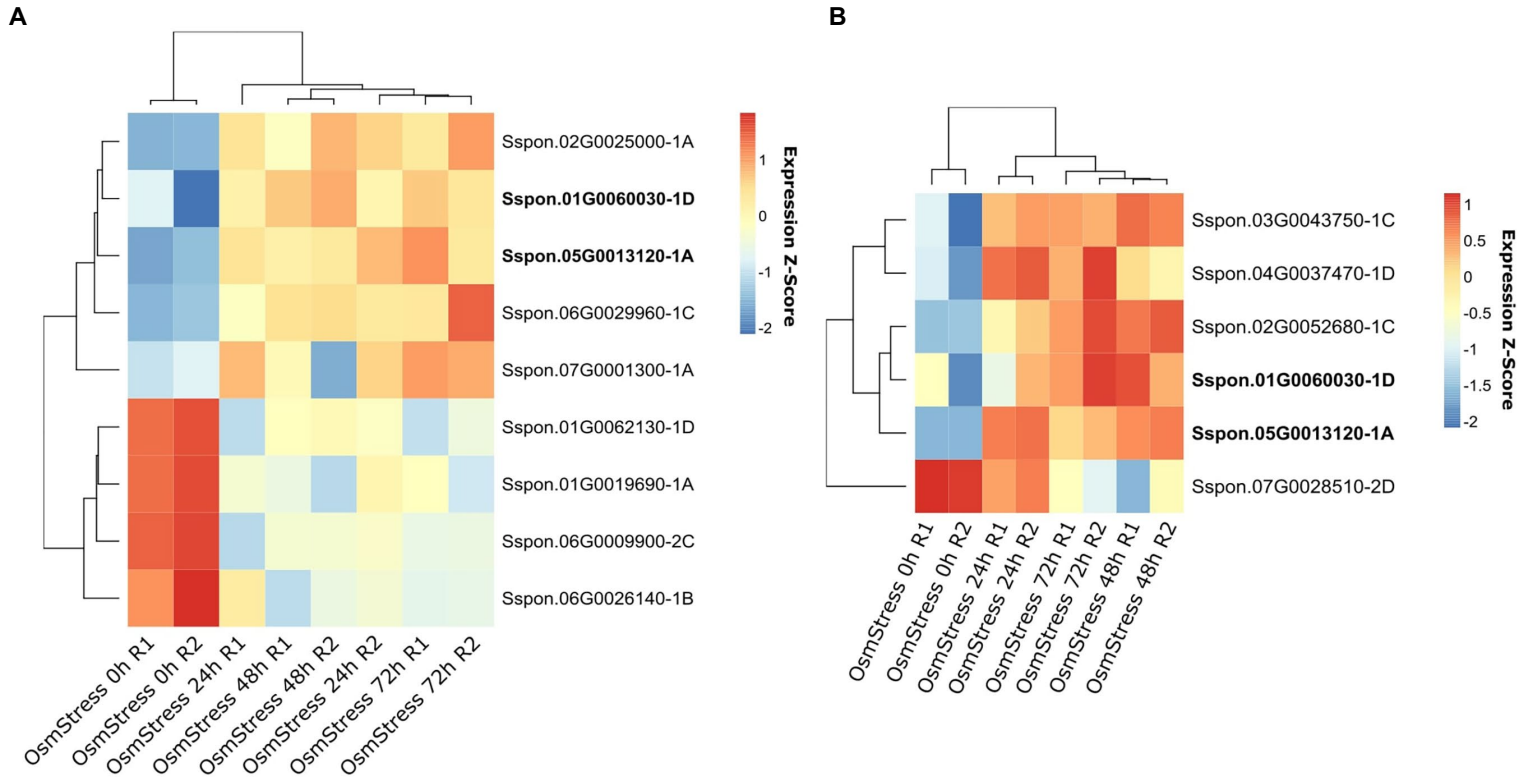
Heatmap of orphan genes (OGs) differentially expressed (DE) under osmotic stress. Plants were subjected to osmotic stress for 24, 48, and 72 h, and other plants were maintained without osmotic stress (0 h). RNA samples were extracted from the leaves **(A)** and roots **(B)** of *S. officinarum*.

In the osmotic stress experiment conducted on leaves and root samples from *S. officinarum*, a total of 4,207 genes were DE in the leaves (1,815 upregulated and 2,392 downregulated), while 4,222 genes were DE in the root samples (1,707 upregulated and 2,515 downregulated). Of these genes, 13 OGs were identified as DE, nine in the leaf samples and six in the root samples. Two OGs, Sspon.01G0060030-1D and Sspon.05G0013120-1A, were detected as DE in both the root and leaf tissues (Figure 4 and Supplementary Table 7).

**Figure 5:**
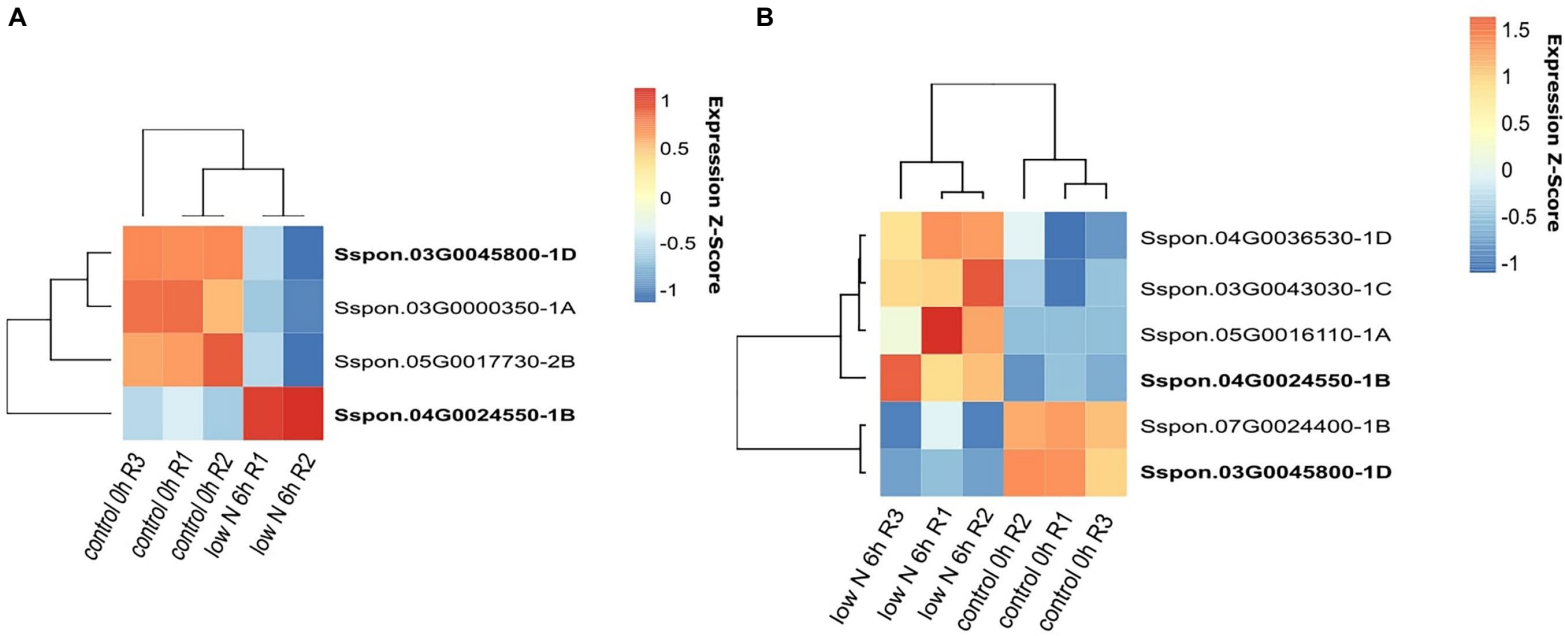
Genes differentially expressed (DE) under low-nitrogen conditions. RNA samples were extracted from leaves of the ROC22 **(A)** and Badila **(B)** genotypes.

In the sugarcane plants exposed to low-nitrogen conditions, most of the DE genes were detected in the leaf samples (4,524 genes in the Badila variety and 2,345 genes in the ROC22 hybrid), while in the roots, 894 and 726 genes were estimated to be DE in Badila and ROC22, respectively. In the root samples, the number of genes upregulated was three times greater than the number of genes downregulated. This pattern was observed in both genotypes. We did not detect DE OGs in the roots of either genotype; however, in the leaves, we detected six DE genes in Badila (four upregulated and two downregulated) and four DE genes in the ROC22 hybrid (one upregulated and three downregulated; Figure 5 and Supplementary Table 7).

### Co-expression Network and Modules With OGs

The co-expression network was built with the full set of sugarcane genes, including 288 OGs with estimated expression values. The sugarcane genes were distributed among 153 modules, of which 78 had at least one OG. More than one-third of the OGs (120 of 319) were assigned to seven modules. An enrichment analysis of these modules containing OGs (Fisher’s exact test; value of *p* ≤ 0.05) suggested that the genes are associated with several biological processes (Supplementary Table 8). Overall, the most frequent Gene Ontology terms observed in modules containing OGs included those associated with responses to several stimuli and defence mechanisms (Supplementary Figure 8). We identified a module with a set of co-expressed genes containing 64 OGs, which were enriched in Gene Ontology function terms related to transport (GO:0006810), response to starvation (GO:0042594), and response to external stimulus (GO:0009605). We also found modules enriched with genes associated with protein modification (GO:0032446; module containing 13 OGs) and regulation of DNA methylation (GO:0044030; 10 OGs) as well as two modules with eight OGs each, which were associated with general processes such as macromolecule metabolic process (GO:0043170) and catabolic process (GO:0009056). Interestingly, a total of 24 OGs DE under stress conditions were included in modules enriched in genes functionally associated with response to stimuli, such as response to nutrient levels (GO:0031667), response to fungus (GO:0009620), and response to stress (GO:0006950).

## Discussion

### Identification and Characterization of Sugarcane OGs

The definition and determination of OGs are context dependent, but generally, a gene that lacks detectable sequence homology in other taxa is typically classified as an OG (Rödelsperger et al., 2019). OGs with no homology to genes in other species are candidates for the *de novo* evolution of genes. However, we cannot reject the hypothesis that, in some cases, these homolog genes were missing in these target species because the genome is incomplete, missing sequences that could be biologically informative, including entire genes. Additionally, most OGs identified in our study did not show sequence similarity to any known functional domain. There are two possible reasons for this, which are linked to OG origin, complete divergence from ancestral sequences or *de novo* emergence from non-genic sequences (Van Oss and Carvunis, 2019; Singh and Syrkin Wurtele, 2020; Vakirlis et al., 2020). In the first scenario, a pair of genes sharing a common ancestor can diverge at some point when similarity is no longer detectable. Because of the lack of similarity with known functional domains, it is also possible that these genes are ncRNAs. Even though we did not observe any OGs similar to ncRNAs deposited in databases, some OGs were predicted to be lncRNAs based on the coding potential calculator. However, this method assumes that a protein-coding RNA is more likely to have a long open reading frame than a non-coding transcript. This likely explains the strong positive correlation that we observed between gene length and the probability of being a coding sequence. Consequently, short OGs tend to be assigned as lncRNAs.

### TEs Might Be Involved in OGs Origin in Sugarcane

The presence of TE fragments within a coding region is not surprising. One of the molecular mechanisms by which TEs are recruited to be part of a gene is named exonization. It takes place after TE insertion into an intron, after which parts of the TE can be incorporated, leading to the presence of a TE exon in a protein-coding gene (Schrader and Schmitz, 2018). TE-derived proteins have been recurrently domesticated during evolution, and they have contributed to adaptive evolutionary innovation (Jangam et al., 2017; Schrader and Schmitz, 2018). In support of this view, a mechanism of OG birth *via* TEs has been reported in rice (Jin et al., 2019). Similarly, most OGs predicted in primates were found to include fragments of TEs in the transcript (Toll-Riera et al., 2008). In the sugarcane genome, we observed that most genes, both orphan and non-orphan, contained TE traces producing poor alignment in the coding region. This may indicate frequent recruitment of TEs as part of novel genes.

### OGs Expression Patterns and Responses to Stress Conditions

The expression profiles of OGs across sugarcane hybrids may indicate that these genes have similar expression patterns when we compare genotypes with the same geographical origin. Indeed, it would be expected that hybrids from the same breeding programme would show greater genetic similarity, including regulatory elements, because of the admixture among genotypes sharing a common parentage. We also observed that the expression pattern of the hybrids was more similar to that of *S. officinarum* than to those of other species, suggesting preserved regulatory control of the expression of these genes after hybridization. These findings also suggest that genotype clustering is influenced by parental genome contributions. Indeed, all modern sugarcane varieties originated from hybridization between *S. officinarum* and *S. spontaneum*, followed by several backcrosses using *S. officinarum* to restore a high sucrose content (Price, 1961). This breeding process resulted in unequal contributions of the sub-genomes in *Saccharum* hybrids. For instance, the genome of the R570 hybrid received approximately 80% of its chromosomes from *S. officinarum* (D’Hont, 2005), which could explain the similar expression patterns between hybrids and *S. officinarum*. Furthermore, the observation that some OGs have tissue-specific expression may indicate a sophisticated mechanism controlling their expression, perhaps mediated by TEs. Previous studies revealed that TEs could contribute to the emergence of new regulatory elements in a tissue-specific manner (Feschotte, 2008; Sundaram et al., 2014; Trizzino et al., 2018), which is a theory based on Barbara McClintock’s discovery that TEs can control gene expression (McClintock, 1956). This is a plausible conjecture because more than 70% of the sugarcane genome is represented by TEs, and fragments of these elements were found in some OGs.

Evidence of OGs being regulated under stress conditions has been reported previously. Studies have demonstrated the importance of lineage-specific genes in plants subjected to biotic and abiotic stresses. For example, in cowpea (*V. unguiculata*), OGs seem to be more involved than conserved genes in drought adaptation, as OG expression was highly induced compared to conserved gene expression under drought conditions (Li et al., 2019). Similarly, the expression levels of two OGs, CpCRP1 and CpEDR1, are modulated when individuals of a model plant widely studied for understanding the mechanism of desiccation are subjected to dehydration and rehydration processes (Giarola et al., 2014). Here, most sugarcane OGs were DE in experiments in which plants were exposed to abiotic stress. However, we cannot confirm that all these genes changed their expression pattern as an adaptive response to these stresses. Further investigation needs to be carried out to experimentally validate the effectiveness of these genes for minimizing stress effects. Even though these findings were not experimentally validated, to confirm that these genes were DE, we observed a significant number of OGs that were up- and downregulated simultaneously in independent genotypes and analyses in the same experiment.

### Modules Containing OGs Are Functionally Enriched With Stress-Response Genes

The functional prediction of OGs based on sequence homology is not possible. Hence, we built a co-expression network to provide some insight into the functionality of these genes. This prediction method follows the guilty-by-association rationale (Zhang and Horvath, 2005), where the functional enrichment of modules containing genes co-expressed with OGs suggests the potential biological roles of those OGs. In general, we observed OGs distributed in several network modules, indicating that these genes are involved in diverse biological activities. This is in accordance with the findings of previous studies that described OGs as being involved in several biological networks (Beike et al., 2014; Giarola et al., 2014; Khraiwesh et al., 2015; Schlötterer, 2015). Accordingly, the main biological function attributed to these genes, the stress response, is itself a complex mechanism involving multiple biological pathways (Shulaev et al., 2008; Mantri et al., 2011). We highlight the evidence that most modules containing OGs were functionally enriched in biological processes related to stimulus responses, including those associated with various stresses. In addition to canonical terms related to stresses, such as defence responses and responses to stimuli, we also detected modules enriched, for example, with genes associated with sulphate transport and responses to auxin. Genes functionally related to these processes play an important role in stress responses (Rahman, 2012; Chan et al., 2013; Shani et al., 2017).

Because sugarcane OGs have no homology with genes in other organisms, we cannot infer their functions based on homology searches. In fact, in most cases, we did not find a known domain in the OG sequences, suggesting that sequence divergence is not the main source of OG origin in sugarcane. Even though we did not obtain strong evidence of their functionality, we cannot discard the relevance of these genes for understanding the unique biological aspects of the *Saccharum* lineage. To advance our knowledge, further investigation needs to be undertaken to better understand how these genes effectively participate in the stress response.

To date, the potential biotechnological applications of a few OGs have been tested. The QQS gene, an *Arabidopsis* gene involved in carbon and nitrogen allocation, was introduced into the soybean genome and increased starch and protein levels in the leaves (Li and Wurtele, 2014; Li et al., 2015; O’Conner et al., 2018). OGs were also validated *via* gene editing as vital for soluble sugar metabolism in brassicas (Jiang et al., 2020). Despite the biological relevance of the OGs, it is still unknown how many of them are functional and produce stable proteins (Schlötterer, 2015; McLysaght and Hurst, 2016). Although OGs are not essential for survival, they may play an important role in responses to environmental stresses (Arendsee et al., 2014; Ma et al., 2020).

In our study, we developed an approach for the identification of OGs in sugarcane and characterization of their expression patterns. We propose that non-coding regions might provide important genetic raw material for the functional innovation, by which novel ORFs are selected and may evolve into adaptive stress response pathways. Finally, the OGs responsive to abiotic stress in sugarcane might be good candidates for further experiments to investigate their biological functions.

## Data Availability Statement

The original contributions presented in the study are included in the article/Supplementary Material; further inquiries can be directed to the corresponding author.

## Author Contributions

CC-S, MM, and DS conducted the experiments. CC-S and AA analysed the data. CC-S wrote the manuscript. All authors discussed the data, interpreted the results, read and edited the manuscript, and approved the final version.

## Funding

This work was supported by grants from the Fundação de Amparo à Pesquisa de Estado de São Paulo (FAPESP, 08/52197–4), Conselho Nacional de Desenvolvimento Científico e Tecnológico (CNPq), and Coordenação de Aperfeiçoamento de Pessoal de Nível Superior (CAPES—Computational Biology Program 88882.160095/2013–01). CC-S received a postdoctoral fellowship from FAPESP (2015/16399–5 and BEPE 2017/26781–0); AA received a PhD fellowship from FAPESP (2019/03232–6); and CS and MM received postdoctoral fellowships from FAPESP (CS 2015/24346–9 and MM 2014/11482–9). AS received a Research Fellowship from CNPq (312777/2018–3).

## Conflict of Interest

The authors declare that the research was conducted in the absence of any commercial or financial relationships that could be construed as a potential conflict of interest.

## Publisher’s Note

All claims expressed in this article are solely those of the authors and do not necessarily represent those of their affiliated organizations, or those of the publisher, the editors and the reviewers. Any product that may be evaluated in this article, or claim that may be made by its manufacturer, is not guaranteed or endorsed by the publisher.

## Footnotes

^1^ http://www.girinst.org/repbase
