# Supplementary material for: Taxonomically Restricted Genes Are Associated With Responses to Biotic and Abiotic Stresses in Sugarcane (Saccharum spp.)
Source: Front Plant Sci. 2022 Jun 30;13:923069. doi: 10.3389/fpls.2022.923069 (PMC9280035; doi:10.3389/fpls.2022.923069)
Supplement: Supplementary file 9 [file Data_Sheet_1.pdf]

1 SUPPLEMENTARY FIGURES

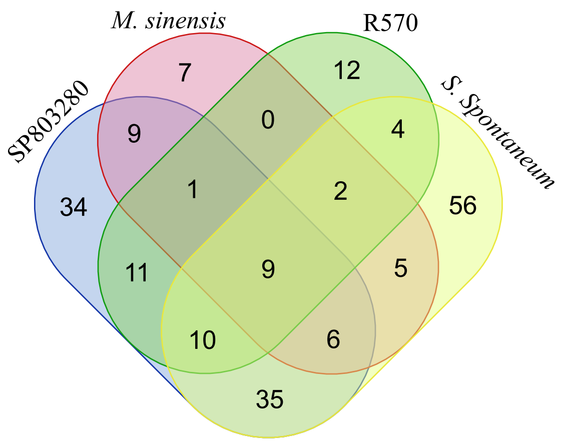

Supplementary Figure 1. Orphan Genes homologs detected in Saccharinae species (*Saccharum* spp. and *M. sinensis*).

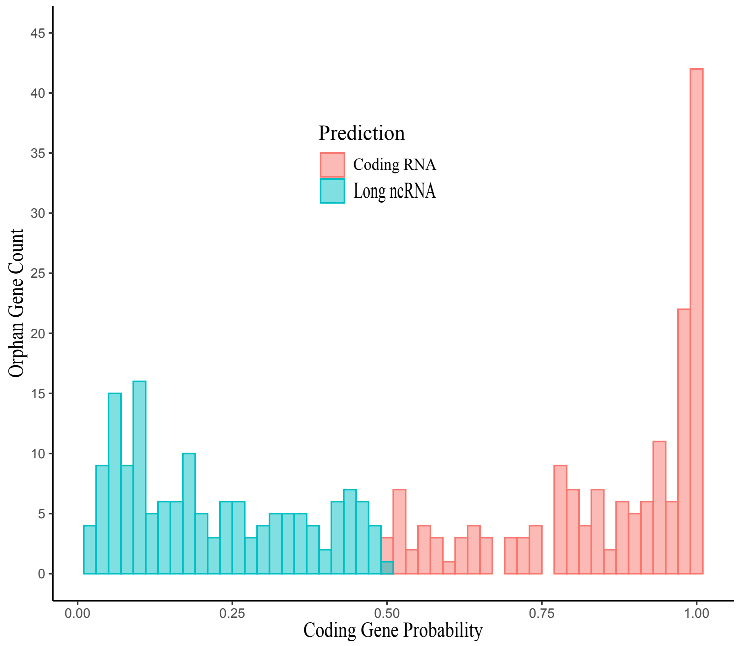

Supplementary Figure 2. Gene count and probability of a given OG to be classified as coding (light red bar) or long non-coding RNA (light blue bar) based on the coding potential calculator (CPC2)

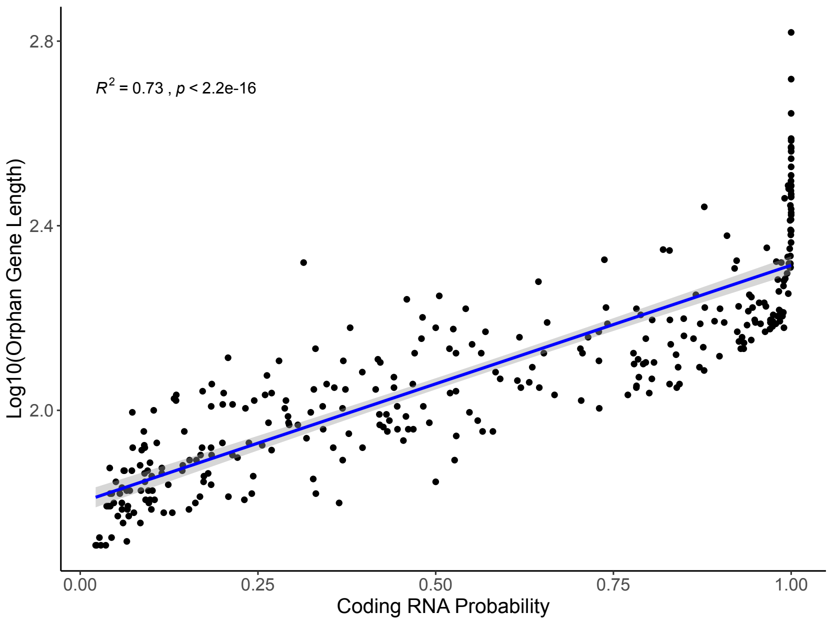

Supplementary Figure 3. Pearson's Correlation Analysis was performed to analyze the correlation between the length of each OGs and the probability of this OGs being classified as a coding RNA.

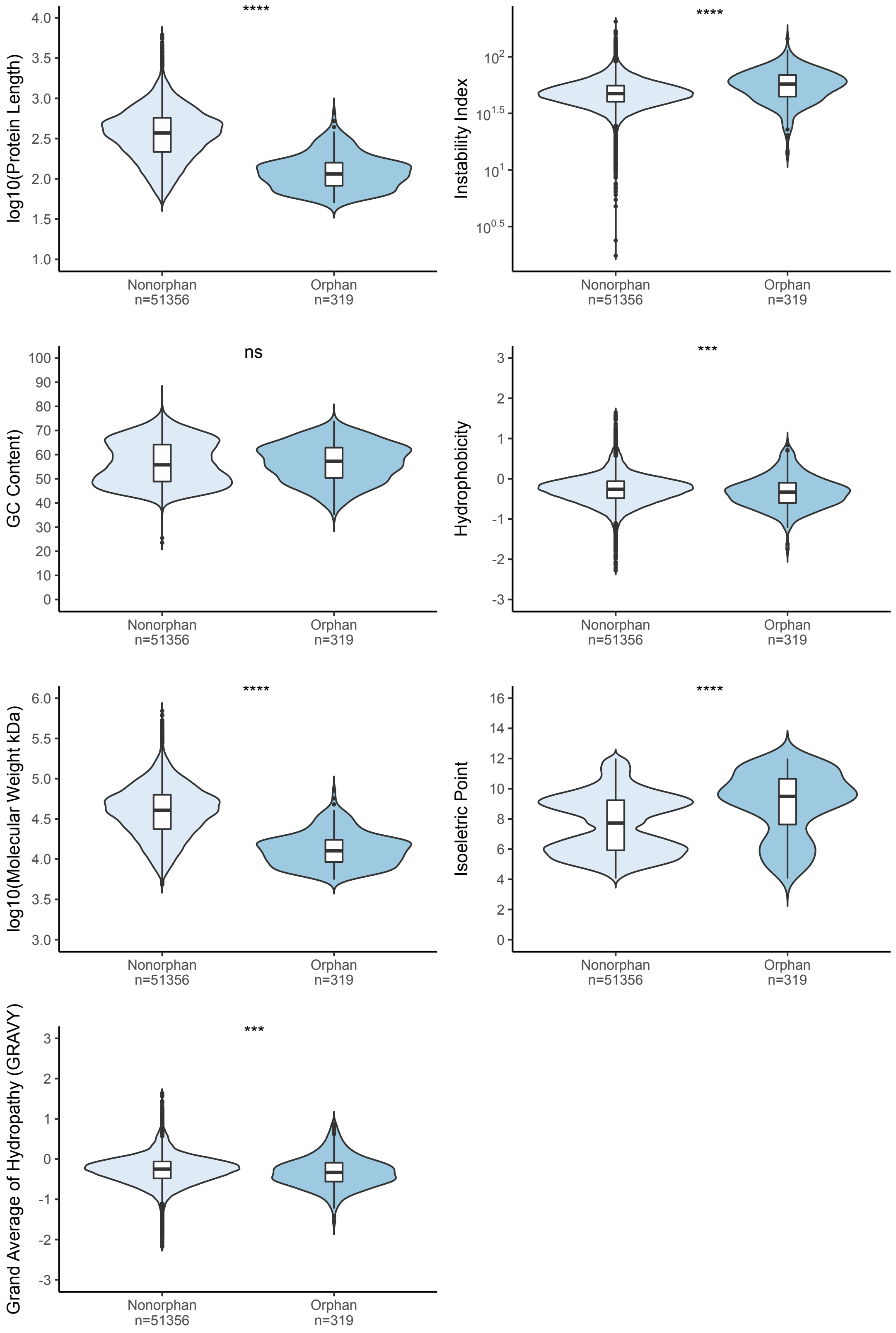

Supplementary Figure 4. Comparison of physical and chemical properties of the OGs and non-OGs. Wilcoxon-Test, significance levels: *ns* no significance, *** $p \leq 0.001$, **** $p \leq 0.0001$.

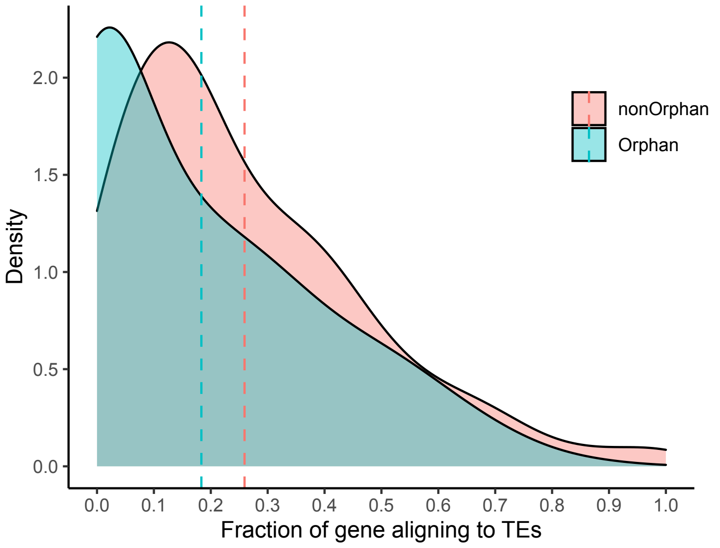

Supplementary Figure 5. Vestiges of TE detected in the genes of *S. spontaneum*. Vertical dashes represent mean value of the alignment fraction

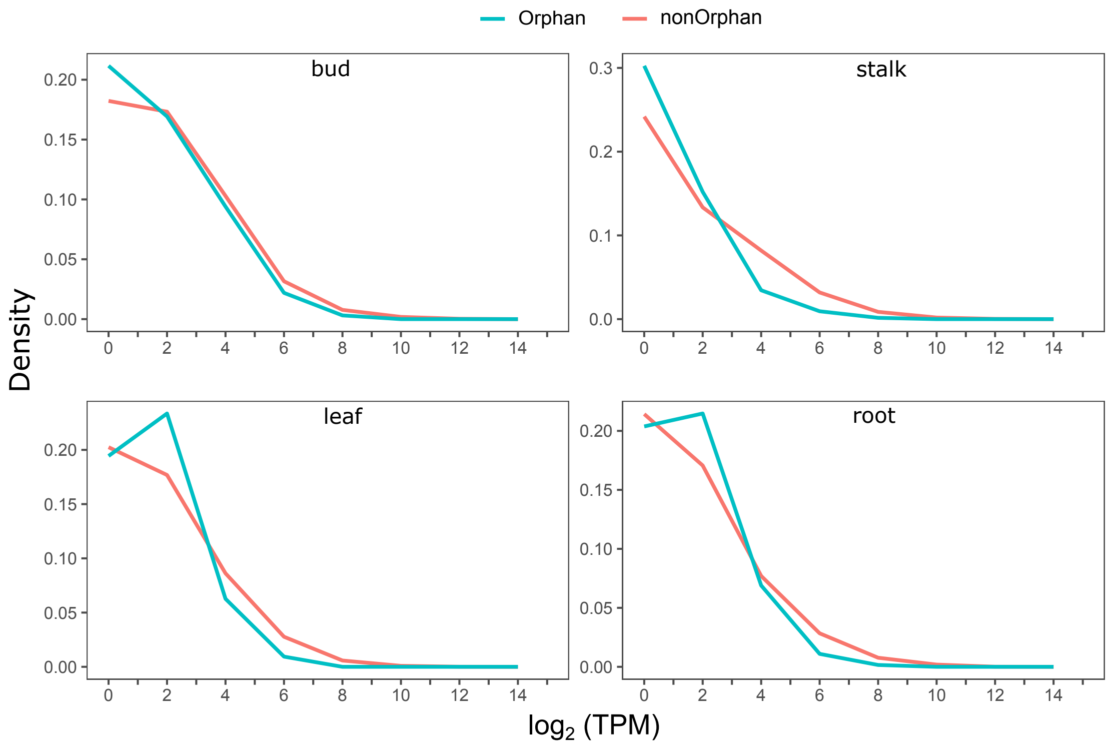

Supplementary Figure 6. Comparison of the expression level of OGs and non-OGs in four sugarcane tissues

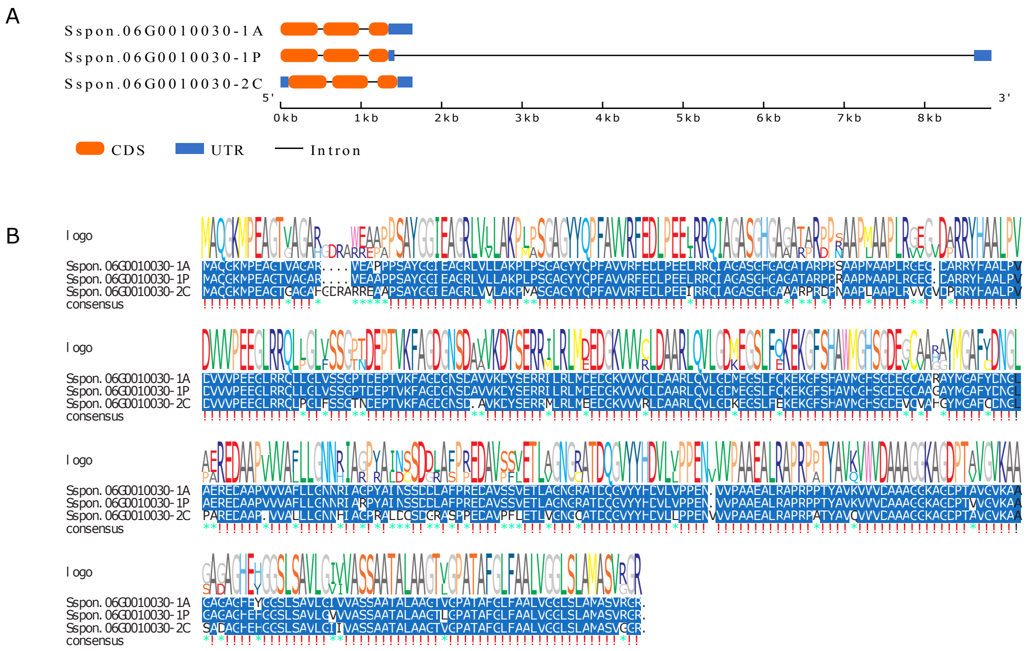

Supplementary Figure 7. Structure (A) and conserved motif (B) of an orphan gene induced under cold stress in two sugarcane genotypes ROC22 and Guitang08-1180. Three copies of this gene were annotated in the *S. spontaneum* genome.

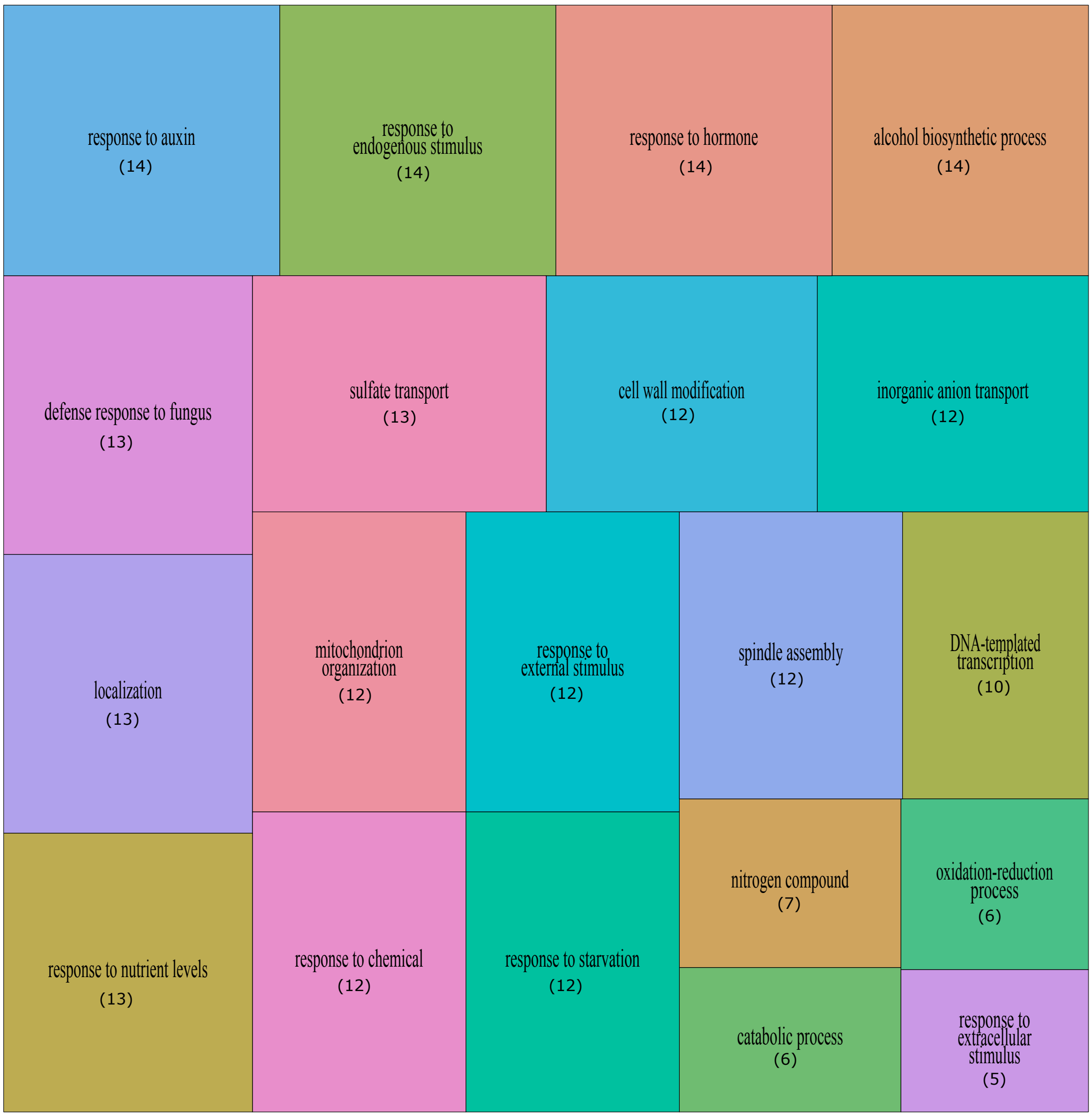

Supplementary Figure 8. Most frequent Gene Ontology terms linked to modules containing orphan genes. GO terms were selected based on enrichment analysis using Fisher exact test (p-value ≤ 0.05). The values between parenthesis correspond to the number of orphan genes associated with the GO term
